# Association of semaglutide with retained gastric contents on endoscopy: Retrospective analysis

**DOI:** 10.1055/a-2550-1468

**Published:** 2025-04-04

**Authors:** Garrick Han Gu, Connor Pauplis, Taylor Seacor, Deepika Devuni, Anita Krishnarao

**Affiliations:** 1 3354 Internal Medicine, UMass Memorial Health, Worcester, United States; 2 12262 Internal Medicine, UMass Chan Medical School, Worcester, United States; 3 12262 Division of Gastroenterology and Hepatology, UMass Chan Medical School, Worcester, United States; 4 3354 Gastroenterology and Hepatology, UMass Memorial Health, Worcester, United States

**Keywords:** Endoscopy Upper GI Tract, Quality and logistical aspects, Sedation and monitoring, Performance and complications

## Abstract

**Background and study aims:**

We investigated the effect of semaglutide, a glucagon-like peptide-1 (GLP-1) agonist therapy, on retained gastric contents during endoscopy through a retrospective case-control study.

**Patients and methods:**

We performed a retrospective case-control study to evaluate the effect of semaglutide on rates of retained gastric contents (RGC) visualized during esophagogastroduodenoscopy (EGD). Cases and controls were matched using multidimensional propensity score matching: age, gender, body mass index, and EGD indication. Pairs were analyzed using McNemar testing and Mann-Whitney non-parametric tests.

**Results:**

Of the patients on GLP-1 therapy at time of EGD, 12.5% had RGC, compared with 1.3% in the control group (confidence interval [CI] 7.2% to 17.7%, *P* < 0.0001). Approximately 23% of patients prescribed GLP-1 therapy for weight loss had RGC at time of EGD compared with the control group (CI 13.4% to 32.6%, *P* < 0.0001). Only 2.6% of patients prescribed GLP-1 therapy for diabetes had RGC at time of EGD compared with the control group (CI -0.9% to 6.1%, *P* = 0.5). Patients receiving GLP-1 therapy with RGC at time of EGD did not differ from non-RGC patients in dosing of GLP-1 agonist ( *P* = 0.23) or duration of GLP-1 agonist use prior to EGD ( *P* = 0.98).

**Conclusions:**

Semaglutide use appears to increase risk of having retained gastric contents visualized during endoscopy. Patients on semaglutide for weight loss appear to have a greater risk of RGC compared with patients on semaglutide for glycemic control. This observation may have clinical implications for management of GLP-1 agonist use prior to endoscopy.

## Introduction

Glucagon-like peptide-1 (GLP-1) agonists are a class of medications that inhibit release of glucagon, stimulate insulin secretion, and slow gastric emptying. These medications are rapidly growing in popularity due to their effectiveness in reducing serum glucose levels and achieving weight loss in patients with type 2 diabetes (T2D). Popular GLP-1 agonists include semaglutide, dulaglutide, exenatide, and liraglutide. Of these agents, only semaglutide and liraglutide are US Food and Drug Administration approved for treatment of weight loss. Semaglutide is considered a long-acting GLP-1 agonist and both oral and subcutaneous formulations currently are available. The side effect profile of GLP-1 agonists is predominantly gastrointestinal in nature due to the mechanism of action, and the most common side effects are nausea, vomiting, and diarrhea 1 . Recent studies have also shown a correlation between gastrointestinal side effects and increased risk of retained gastric contents (RGC) during endoscopy (PR = 16.5; 95% confidence interval [CI] 9.08–34.91) 2 . Presence of RGC is problematic because it impairs visualization, impedes interventions, and is associated with increased risk of aspiration events 3 4 .

With the rising popularity of these agents, there has been concern regarding adverse events related to delayed gastric motility. Sodhi et. al. found that use of GLP-1 agonists for weight loss was associated with an increased risk of gastroparesis and bowel obstruction 5 . A variety of case reports have also emerged in recent years describing intraoperative aspiration events in patients on semaglutide 6 7 8 9 . The American Society of Anesthesiologists (ASA) Task Force on Preoperative Fasting, therefore, issued a recommendation in July 2023 to hold GLP-1 agonists the day of an elective procedure if the patient is on daily dosing or for the week of if the patient is on weekly dosing 10 . The ASA has also recommended delaying elective procedure for patients experiencing gastrointestinal symptoms on GLP-1 agonists to minimize potential risk of aspiration 10 .

The American Gastroenterological Association (AGA) subsequently issued a rapid clinical practice update in November of 2023 advocating for an individualized approach to management of GLP-1 agonists prior to endoscopy 11 . The AGA suggests proceeding with upper and/or lower endoscopy if the patient has followed standard pre-procedure fasting instructions and does not have nausea, vomiting, dyspepsia, or abdominal distension 11 . For those with symptoms suggestive of RGC who may experience negative clinical effects if endoscopy is delayed, rapid sequence intubation can be considered, although it may not be feasible in many outpatient-based endoscopy settings 11 . The AGA also has suggested placing patients on a liquid diet 1 day before endoscopy in lieu of stopping GLP-1 agonists 11 . The AGA has also emphasized the importance of considering the indication for GLP-1 agents because risk of stopping GLP-1 agonists for weight loss may be less than risk of GLP-1 agonist discontinuation in patients with diabetes. Various other societies have similarly issued guidelines regarding use of GLP-1 agonists prior to procedures 12 13 .

Despite the recent emergence of peri-procedure guidelines, there is a definitive lack of high-level evidence related to definitive endoscopic risk related to GLP-1 agonist use. Therefore, we performed a retrospective cohort study to elucidate the relationship between GLP-1 agonist use and presence of RGC during esophagogastroduodenoscopy (EGD). Semaglutide was chosen as the GLP-1 agonist of focus due its efficacy in treating weight loss and T2D and its popularity with rapid increase in prescription rates at the time the study was designed 14 15 .

## Patients and methods

A total of 1045 patients on semaglutide with an identified history of EGD in the UMass Memorial Healthcare system from March 1, 2022 to August 28, 2023 were identified with SlicerDicer on Epic, using CPT codes ranging from 43180 to 43278. The study end date was chosen because the study site instituted a new protocol to hold all GLP-1 agonists 1 week prior to procedures in September 2023. Of the 1045 identified patients, 893 patients were excluded due to the following criteria: patients who had previously been on any other GLP-1 agonists, patients who received their endoscopy before initiating semaglutide, patients who were critically ill at the time of their endoscopy, patients who held their semaglutide 1 week prior to endoscopy, patients with acute upper gastrointestinal tract bleeding as the primary indication for EGD, and any prior diagnosis of gastroparesis or delayed gastric emptying. All patients in this study fasted at midnight prior to the procedure. A total of 295 referents were identified in the excluded patients who met all criteria and had undergone EGD in the study period prior to initiating semaglutide.

Index and reference groups were then matched using multidimensional propensity score matching utilizing the following: age, gender, body mass index (BMI), and EGD indication. Propensity score matching is a type of observational statistical analysis that attempts to match characteristics of the index and reference groups to reduce bias. By matching subjects by these characteristics, this method can control for these characteristics as possible confounders. These characteristics were chosen due to author assumptions about the possible effect of age, gender, and BMI on baseline gastric emptying, as well as possible unrelated risk of RGC in patients who had EGD for certain indications such as nausea. Propensity score matching was performed with XLStat (Data Analysis and Statistical Solution for Microsoft Excel, Lumivero, Denver 2023), using a Mahalanobis 1:1 match. A total of 152 index-referent pairs could be identified using the propensity score matching system. It should be noted that restricting the referent sample size to 152 through this method attempted to control for possible confounders, but did reduce statistical power and increase risk of selection bias. However, it was felt that the advantage of controlling for these confounders outweighed loss of statistical power.

Pairs were analyzed using McNemar testing to determine presence of RGC on EGD procedure result notes. RGC was defined as any amount of solid food present within the stomach based on clinical evaluation by the endoscopist, and therefore, indicated in their procedure note. Dose, presence of neuropathy, or duration of GLP-1 therapy prior to EGD in patients were analyzed using Mann-Whitney non-parametric tests.

## Results

Patients were evenly distributed among index and reference groups, with no statistically or clinically significant differences in age, ethnicity, BMI, cirrhosis, or current prescriptions for gastrointestinal slowing medications such as tricyclic antidepressants, opioids, calcium channel blockers, muscarinic receptor antagonists, octreotide, phenothiazines, and cyclosporine 16 17 . There was a statistically significant difference between index and referents in A1c ( Table 1 ), and mean time between GLP-1 agonist initiation and endoscopy was 4.7 months. The most common EGD indication was gastroesophageal reflux disease, followed by dysphagia and dyspepsia.

**Table:** **Table 1** Demographics of study population.

	GLP-1 patients (n = 152)	Controls (n = 152)	*P* value
Mean age (years) (mean ± SD	57.8 ± 11.8	57.2 ± 12.8	0.5
White patients [n (%)]	125 (82%)	125 (82%)	0.96
Mean BMI (kg/m ^2^ ) (mean ± SD)	34.4 ± 6.9	35.5 ± 6.6	0.06
Gastrointestinal slowing medications (%)	15 (9.8%)	20 (13.3%)	0.62
A1c (mean ± SD)	6.07 ± 2.2	5.95 ± 1.7	0.01
History of cirrhosis [n (%)]	15 (9.8%)	11 (7.2%)	0.57
Mean time GLP1 start until endoscopy (months ± SD)	4.7 ± 4.0		
Dose (mean mg/week ± SD)	0.83 ± 0.57		
Indication for GLP1 = weight loss [n (%)]	74 (48.6%)		
Indication for EGD			
GERD	58 (40%)	61 (38%)	
Dysphagia	16 (11%)	22 (15%)	
Dyspepsia	14 (9%)	18 (12%)	
Other	61 (40%)	54 (35%)	
BMI, body mass index; GLP-1, glucagon-like peptide-1; EGD, esophagogastroduodenoscopy; GERD, gastroesophageal reflux disease; SD, standard deviation.			

**Fig. 1:**
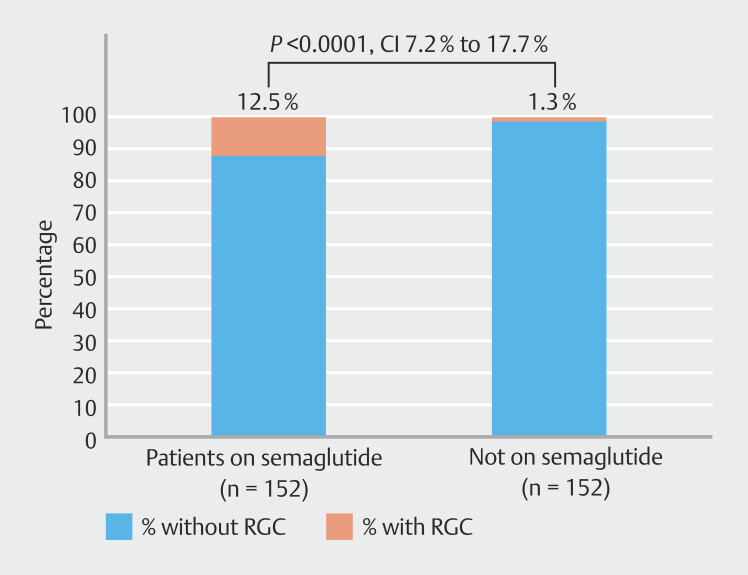
Percentage of total patients in study with retained gastric contents (RGC) on upper endoscopy (EGD). Patients on semaglutide demonstrated greater rates of RGC ( *P* < 0.0001).

**Fig. 2:**
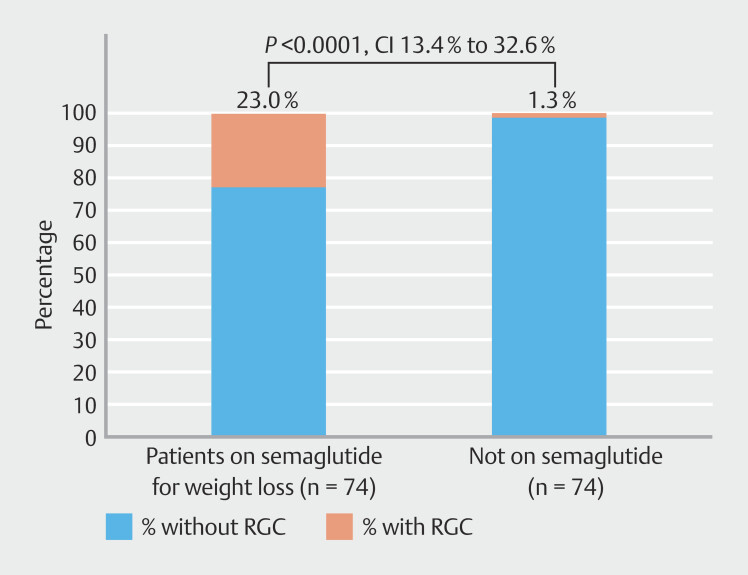
Percentage of patients taking semaglutide for weight loss with retained gastric contents (RGC) on upper endoscopy (EGD). Patients on semaglutide for weight loss demonstrated greater rates of RGC compared with the reference group ( *P* < 0.0001).

**Fig. 3:**
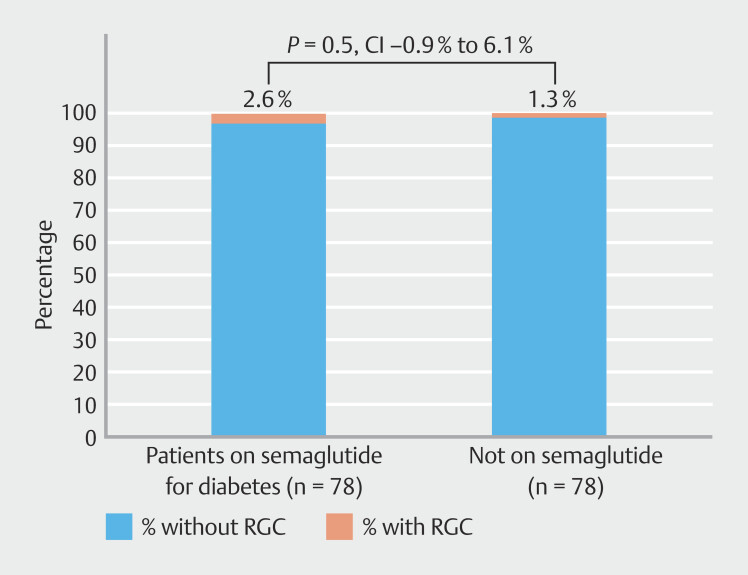
Percentage of Patients taking semaglutide for diabetes with retained gastric contents (RGC) on upper endoscopy (EGD). Patients on semaglutide for diabetes did not demonstrate greater rates of RGC compared with the reference group ( *P* < 0.0001).

**Fig. 4:**
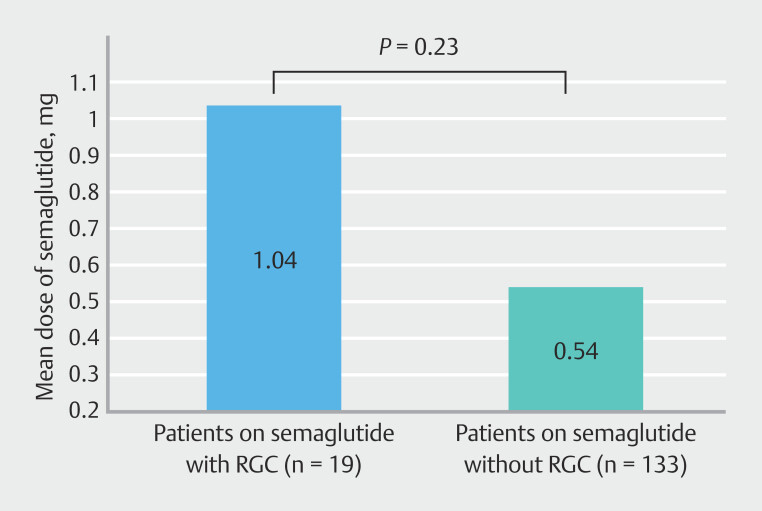
Comparison of dose of semaglutide in patients on semaglutide. Patients who showed RGC were not taking significantly more semaglutide compared with the reference group ( *P* = 0.23).

**Fig. 5:**
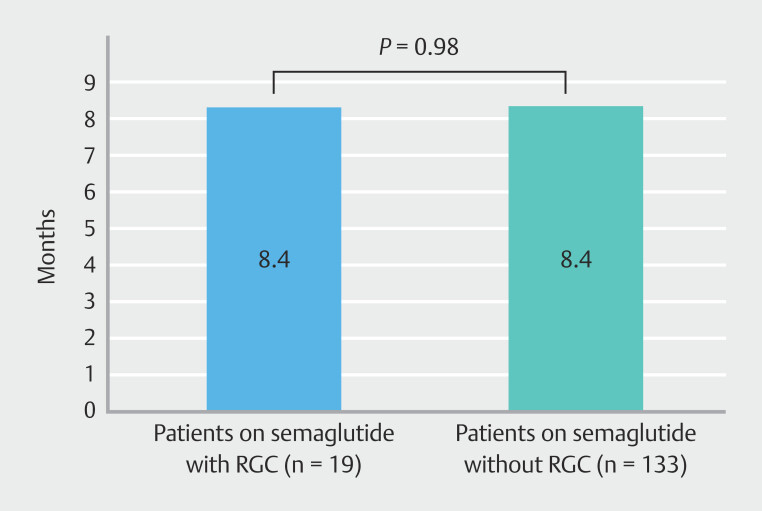
Comparison of duration of semaglutide therapy in patients on semaglutide. There was no difference in duration of therapy of semaglutide in patients who developed RGC compared with the reference group ( *P* = 0.98).

**Fig. 6:**
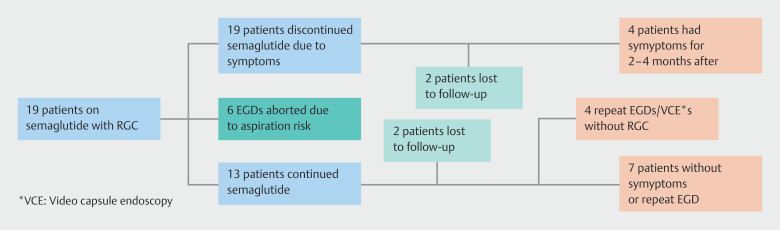
Complications analysis of 19 semaglutide patients that developed RGC.

In the index cohort, 74 patients (48.6%) had been prescribed GLP-1 agonist therapy primarily for weight loss and 78 (51.4%) for diabetes. Approximately 12.5% of patients on GLP-1 therapy at time of EGD had RGC, compared with 1.3% in the reference group ( Fig. 1 ) (95% CI 7.2%-17.7%, *P* < 0.0001). Approximately 23% patients prescribed GLP-1 therapy for weight loss had RGC at time of EGD compared with 1.3% in the reference group ( Fig. 2 ) (95% CI 13.4%-32.6%, *P* < 0.0001). Approximately 2.6% of patients prescribed GLP-1 therapy primarily for diabetes had RGC at time of EGD compared with 1.3% in the reference group ( Fig. 3 ) CI –0.9% to 6.1%, *P* = 0.5). Patients receiving GLP-1 therapy with RGC at time of EGD did not differ from non-RGC patients in either dose ( Fig. 4 ) *P* = 0.23) or duration of GLP-1 therapy prior to EGD ( Fig. 5 ) ( *P* = 0.98). A complications analysis was done on the 19 patients on GLP-1 therapy who demonstrated RGC on EGD ( Fig. 6 ) and six endoscopies were notably aborted due to clinically significant aspiration risk related to a large volume of RGC. All aborted cases had no mention of noncompliance with institutional fasting guidelines. Six of 19 patients with RGC cases later discontinued GLP-1 therapy after EGD, four of whom continued to have gastrointestinal-related symptoms for 2 to 4 months. Of the remaining 13 patients who continued the medication, 11 did not have documented gastrointestinal-related side effects, of whom four patients had a follow-up EGD or video capsule endoscopy that showed no evidence of RGC.

## Discussion

This retrospective cohort study focused on the relationship between GLP-1 agonist use and presence of RGC during EGD. This is one of the few studies to date evaluating endoscopic data for RGC as it pertains to GLP-1 agonist therapy. Although several studies have attempted to establish this relationship using gastric emptying studies, few studies have documented endoscopic evidence of RGC and results have varied 2 18 19 . Furthermore, this is the first study solely focused on use of semaglutide. Semaglutide is the most popular GLP-1 agonist prescribed in the United States with more than 8 million prescriptions in 2021 20 . The focus on semaglutide in this study, therefore, was intentional because it represents an archetypal long-acting GLP-1 agonist with varying indications including weight loss and glycemic control in diabetes.

Results of this study highlighted a near tenfold increase in RGC in patients taking GLP-1 agonists compared with matched referents, which may have clinical significance due to the effect size. Furthermore, results showed that the specific indication for use of semaglutide had a statistically significant effect on presence of RGC. Study participants taking GLP-1 therapy for weight loss purposes were statistically more likely to have RGC noted on endoscopy, whereas those taking semaglutide for diabetes did not show a difference from referents. These data support previous literature from Nakatani et al, which demonstrated that the impact of GLP-1 agonist on gastric and small intestinal emptying via capsule endoscopy was significantly different in those with and without diabetic neuropathy (DN) (0:48:00 h in patients without DN vs 1:12:36 ± 1:04:30 h in patients with DN) 21 . This effect may be explained by preexisting gastric emptying delay prior to administration of GLP-1 agonist therapy in those with DN. Proposed mechanisms include reduced gastric dilation in the proximal gastric region, decreased gastric emptying in the pyloric region, and greater pyloric motor activity in the diabetic population 21 . Findings from this study indicate that diabetes may be protective against GLP-1-associated delay on gastric emptying, although further studies would be needed to corroborate this finding. It is also worth noting that this study did not show a statistical difference in RGC rates between patients on different doses of GLP-1 therapy. However, patients with identifiable endoscopic RGC were on an average 93% higher dose than those without RGC in this study. Our data, therefore, are skewed toward the possibility that a study with greater power may demonstrate an effect of GLP-1 agonist dose on likelihood of RGC. From a clinical perspective, this would allow for risk stratification for aspiration based on indication for GLP-1 agonist use as well as GLP-1 dose at time of EGD or other procedures.

Complications analysis of these data elucidated additional trends. In a small subset of patients, discontinuing GLP-1 therapy as a result of gastrointestinal side effects did not achieve resolution of symptoms until 2 to 4 months following drug discontinuation. This effect cannot be adequately explained with pharmacokinetics that demonstrate a 1-week half-life of semaglutide 22 . Although there were no repeat endoscopic studies to support a correlation between persistent RGC and symptom profile, this information may be clinically relevant when advising patients about gastrointestinal side effects of semaglutide. Although not commented on in the results, it is also worth noting that two of the patients with RGC on initial EGD underwent subsequent EGD while remaining on GLP-1 therapy but were instructed to follow a gastroparesis diet and remain without oral intake for 24 hours prior to EGD. Repeat EGD in these two patients ultimately did not show RGC despite the fact that they remained on GLP-1 therapy, which suggests that pre-procedure recommendations could potentially be directed toward dietary modification and/or duration of fasting period prior to procedures rather than discontinuation of GLP-1 therapies.

Given the surge in GLP-1 agonist use in treatment of diabetes and obesity, further prospective studies are needed to investigate the impact of GLP-1 agonists on RGC rates based on therapeutic indication, which can ultimately help guide endoscopy-related and peri-procedure recommendations. In addition, there is a paucity of data about the likely similar impact of newer dual and triple agonist therapies such as tirzepatide and retratutide, which include GLP-1 agonists. These agents could also be evaluated, ideally in a prospective manner, to similarly evaluate risk profiles related to procedures. Lastly, dose-dependent effects of GLP-1 agonists on RGC require further investigation because data from our study point toward a possible correlation. The RGC patient sample size was insufficient to determine statistical significance, but the two-fold difference in absolute value compared with non-RGC patients may possibly suggest a correlation between dose of semaglutide and risk of delayed gastric emptying.

Our study has several strengths worth noting, including standardized data collection using a clinically relevant measure of endoscopic data. In addition, by using propensity score matching, we attempted to eliminate possible unmeasured confounders including medications commonly implicated in gastric dysmotility. The findings from this study should be considered in light of several limitations. Due to strict exclusion criteria, the patient population was limited to 155 patients at a single large academic center, which may limit generalizability of results. The study was underpowered to detect important relationships such as dose-dependent effects on our primary outcome. Data also relied on endoscopist reporting of RGC on procedure reports as a qualitative measure, which introduces potential bias because that was not reported in a standardized manner.

## Conclusions

In conclusion, our real-world retrospective cohort study suggests that GLP-1 agonists are associated with a higher incidence of RGC on EGD and that the indication for GLP-1 agonist use has a profound effect on rates of RGC. Our study demonstrated that individuals on GLP-1 therapy for weight loss had higher rates of RGC documented on EGD than individuals on GLP-1 therapy for glycemic control. Therefore, these study results provide clinicians with a valuable risk stratification tool for assessing aspiration risk in patients undergoing endoscopic procedures and likely also surgery.
